# Conservation and Diversity of miR166 Family Members From Highbush Blueberry (*Vaccinium corymbosum*) and Their Potential Functions in Abiotic Stress

**DOI:** 10.3389/fgene.2022.919856

**Published:** 2022-05-16

**Authors:** Yuening Li, Xianglong Wang, Qingxun Guo, Xinsheng Zhang, Lianxia Zhou, Yang Zhang, Chunyu Zhang

**Affiliations:** ^1^ College of Plant Science, Jilin University, Changchun, China; ^2^ Helong Forestry Co., Ltd, Changbai Mountain Forest Industry Group, Yanji, China

**Keywords:** miR166, highbush blueberry, evolutionary conservation, functional diversification, abiotic stress, target genes

## Abstract

MicroRNA166 (miR166) is highly conserved and has diverse functions across plant species. The highbush blueberry (*Vaccinium corymbosum*) genome is thought to harbor 10 miRNA166 loci (*Vco-miR166*), but the extent of their evolutionary conservation or functional diversification remains unknown. In this study, we identified six additional *Vco-miR166* loci based on conserved features of the miR166 family. Phylogenetic analyses showed that mature Vco-miR166s and their precursor cluster in several clades are evolutionary conserved with diverse species. The *cis*-regulatory elements in the *Vco-miR166* promoters indicated functions related to different phytohormones and defense responses. We also identified putative targets of vco-miR166s, which targeted the same gene families, suggesting the functional conservation and diversification of *Vco-miR166* family members. Furthermore, we examined the accumulation patterns of six mature Vco-miR166s in response to abiotic stresses by stem-loop reverse RT-qPCR, which revealed their upregulation under freezing, cold, and heat stress, while they were downregulated by drought compared to control growth conditions. However, *Vco-miR166* members showed different expression patterns when exposed to salt stress. These results showed that conserved Vco-miR166 family members display functional diversification but also coordinately influence plant responses to abiotic stress.

## Introduction

MicroRNAs (miRNAs) are small [20–24 nucleotides (nt)] non-coding regulatory RNAs derived from longer precursor (pre-miRNA) molecules with stem-loop structures. The pre-miRNA is processed by DICER LIKE1 (DCL1) into the mature miRNA, which then interacts with its target transcripts. Mature miRNAs mediate almost all plant cellular and metabolic processes by regulating gene expression at the posttranscriptional level (Thakur et al., 2011; Fang and Wang, 2021).

In plants, miR166 is a highly conserved miRNA implicated in a wide range of cellular and physiological processes by regulating its cognate target genes encoding members from the homeodomain leucine zipper (HD-ZIP III) family of transcription factors (Miyashima et al., 2013; Singh et al., 2014; Yadav et al., 2021). Recent evidence showed that the miR166 family plays crucial roles in response to abiotic stress. In mulberry (*Morus multicaulis*), miR166f targets the transcripts encoding an HD-ZIP III transcription factor whose expression is induced by drought stress and is a positive regulator of drought stress tolerance (Li R. et al., 2018). In Arabidopsis (*Arabidopsis thaliana*), multiple miR166 members accumulate when seedlings are exposed to low temperature (Zhou et al., 2008). In potato (*Solanum tuberosum*), the levels of miR166 family members were downregulated by 4°C treatment but were upregulated by salt stress (Kitazumi et al., 2015; Ou et al., 2015). In barley (*Hordeum vulgare*), miR166 was induced in leaves upon dehydration stress (Kantar et al., 2010). In wild emmer wheat (*Triticum turgidum*), miR166 was downregulated by drought stress (Kantar et al., 2011). In soybean (*Glycine max*), miR166 members play an important role in response to cold, drought, and salinity stress (Li et al., 2017).

Blueberries (*Vaccinium* spp.) are an economically important small fruit crop. The main distribution of commercial blueberries from northern to southern China comprises lowbush blueberry (*V. angustifolium*), highbush blueberry (*V. corymbosum*), and rabbiteye blueberry (*V. ashei*). The complement of miRNAs has been identified in blueberries using high-throughput sequencing. For example, 412 conserved miRNAs belonging to 29 families were identified in fruits from rabbiteye blueberry (Yue et al., 2017); similarly, 127 known and 101 novel miRNAs were identified from flowers, young fruits, and ripe fruits of rabbiteye blueberry (Li G. et al., 2018). In highbush blueberry, 84 known miRNAs and 16 novel miRNAs were identified from fruits, with 10 miRNAs from miR166 family (Hou et al., 2017). However, the function of miR166s in highbush blueberry fruits and the genomic sequence of *miR166* loci are unknown. In particular, the response of the highbush blueberry *miR166*s to abiotic stress has not been reported.

As mature miRNAs are highly conserved within the same family, sequence prediction allows the identification of multiple members of a given miRNA family, including miR166 (Li et al., 2017). The sequence and assembly of the highbush blueberry genome were recently completed, opening the doors to a systematic identification of miRNAs and their precursors in this species (Colle et al., 2019). In this study, we predicted the sequences of miRNA166 family members, their precursors, and their encoding genes by mining the highbush blueberry genome sequence. We demonstrated the evolutionary conservation and functional diversification of this family based on a sequence analysis of mature Vco-miR166s, *Vco-miR166* loci, and *Vco-miR166* promoters and predicted vco-miR166 target genes. We also examined the accumulation patterns of six Vco-miR166s in response to abiotic stresses. The results help elucidate the functions of the miR166 family in highbush blueberry.

## Materials and Methods

### Plant Materials and Stress Treatments

One-year-old plants from the highbush blueberry cultivar ‘Bluecrop’, grown in a growth chamber, were used for salt, drought, freezing, cold, and heat treatments. Plants were also maintained in tissue culture on modified woody plant medium with Murashige and Skoog (MS) vitamins, 1.0 mg/L *trans*-zeatin, and 3% (w/v) sucrose. Plants were subcultured every 28 d and were used for drought stress. All plants used for stress treatments were grown at 25°C under a 16-h-light/8-h-dark photoperiod. Plants were exposed to −5°C for 0, 0.5, 1, 2, 4, or 6 h for freezing treatment; 4°C for 0, 2, 4, 6, 9, 12, for 24 h as cold treatment; and 40°C for 0, 0.5, 1, 1.5, 2, and 3 h as heat treatment. Plants were watered with a 200 mM NaCl solution for 0, 1, 2, 6, 9, 12, or 24 h as salt treatment. For drought treatment, plants in tissue culture were transferred to medium (overlaid with 20% (w/v) PEG-6000 for 1 day, and then PEG-6000 was removed) for 0, 1, 2, 6, 9, 12, and 24 h. For all treatments, the 0-h treatment served as the control. Leaves were collected and immediately frozen in liquid nitrogen for microRNA extraction.

### Identification of Novel MiR166s in Highbush Blueberry

The precursor sequences of miR166 from sweet orange (*Citrus sinensis*), grape (*Vitis vinifera*), and apple (*Malus domestica*) were downloaded from the miRbase database (https://www.mirbase.org) and used as a query against the *V. corymbosum* cv. Draper v1.0 genome scaffolds database (https://www.vaccinium.org/). Homologs were screened as previously described (Li et al., 2017; Hou et al., 2017). The secondary structures and the negative minimal folding free energy (MFE) of each miRNA were assessed and computed using the RNAfold WebServer (http://rna.tbi.univie.ac.at/cgi-bin/RNAWebSuite/RNAfold.cgi) (Mathews et al., 2004; Gruber et al., 2008). The minimal folding free energy index (MFEI) was calculated with the following equation: MFEI = [(MFE/length of the RNA sequence) *100]/(G + C)%. The candidates were then confirmed by comparison with miR166s from other species using the NCBI database (https://www.ncbi.nlm.nih.gov). The mature miR166s of highbush blueberry were predicted by searching against the miRbase database using the precursor sequences of miR166 of highbush blueberry.

### Phylogenetic Analysis and Sequences Alignment

Phylogenetic trees were separately constructed using the UPGMA method with the MEGA X program from miR166 precursors and mature miR166s from highbush blueberry (Vco-miR166s), grape (Vvi-miR166s), sweet orange (Csi-miR166s), and apple (Mdm-miR166s) (Kumar et al., 2018). Sequence alignments were performed using DNAMAN software, version 8.192.

### Promoter Analysis of *Vco-miR166*s

The upstream sequences of *Vco-miR166*s were retrieved from the highbush blueberry genome (https://www.vaccinium.org/). The transcription start sites (TSSs) and TATA-box of *Vco-miR166*s were predicted by TSSP software in Softberry (http://linux1.softberry.com/berry.phtml?topic=tssp&group=programs&subgroup=promoter). About 1,600 bp of sequence upstream of the TSS was analyzed for the presence of known *cis*-acting regulatory sequences held in the PlantCARE database (http://bioinformatics.psb.ugent.be/webtools/plantcare/html/).

### Prediction and Functional Analysis of Vco-miR166 Targets

The target sequences of highbush blueberry Vco-miR166s were predicted with the psRNATarget program (http://plantgrn.noble.org/psRNATarget/) against American cranberry (*Vaccinium macrocarpon*) transcripts (Polashock et al., 2014; Dai et al., 2018). The obtained target sequences were then used to query target genes in the *V. corymbosum* cv. Draper v1.0 genome transcript database (Colle et al., 2019). The functions of Vco-miR166s target genes were predicted by BLAST against the Swiss-Prot database (https://ww.uniprot.org). Genetic similarity and genetic distance were calculated by DICE and Nei’s similarity coefficient, respectively. The phylogenetic tree was constructed using the UPGMA method with NTSYSpc-2.11 F software.

### Reverse-Transcription Quantitative PCR (RT-qPCR)

The microRNAs were extracted using an EASYspin Plant microRNA rapid extraction kit (Aidlab, Beijing, China) from control and abiotic stress samples. Reverse transcription and qPCR were performed with the Mir-X miRNA qRT-PCR TB Green Kit (Takara Inc., Dalian, China) using specific stem-loop RT and forward RT-qPCR primers. qPCR was conducted using the ABI StepOnePlus Real-Time PCR System using *U6* as the reference. Primer sequences are listed in Supplementary Table S1.

## Results

### Identification and Characterization of Vco-miR166s in Highbush Blueberry

Based on the conserved features shared by pre-miR166s sequences from sweet orange (*Citrus sinensis*), grape (*Vitis vinifera*), and apple (*Malus domestica*), we performed a BLAST search of the highbush blueberry genome. We identified three new pre-miR166s, designated here Vco-miR166g, h, and i, following a previous report (Hou et al., 2017) (Table 1). The length of these three new pre-miRNAs ranged from 107 to 186 nt, with three to four predicted mismatches between the 5′ and 3′ arms of the miRNA (Supplementary Figure S1). The minimal folding free energy (MFE) of these new mature Vco-miRNAs was lower (from −67.9 to −43.30) and their MFE index (MFEI) (from 0.85 to 1.08) was higher than those of transfer RNAs (tRNAs), ribosomal RNAs (rRNA), and messengers RNAs (mRNAs). These results suggest that these predicted precursors may be *bona fide* precursors of highbush blueberry miRNAs.

**TABLE 1:** Characteristics of three newly identified miR166 precursors in highbush blueberry.

Locus ID	Length (nt)	NM^a^	MFE^b^ kcal/mol	MFEI^c^	Sequence region on Draper genome	Scaffold_ID on Draper genome	Sequence comparison with similar species in NCBI			
							Accession	Species	Name	Identity (%)
*Vco-miR166g*	107	3	−45.30	0.85	9,638,758–9,638,864	VaccDscaff1	KT004773	*Camellia sinensis*	*Csi-miR166g*	90
*Vco-miR166h*	114	3	−51.70	1.08	37,329,235–37,329,348	VaccDscaff19	NR_127759	*Vitis vinifera*	*Vvi-miR166g*	90
*Vco-miR166i*	186	4	−67.9	0.89	28,809,023–28,809,208	VaccDscaff33	NR_107,986	*Solanum lycopersicum*	*Sly-miR166b*	63

a NM, number of mismatches between predicted 3′ and 5′ arms of the miRNA.

b Minimal folding free energy.

c Minimal folding free energy index.

BLAST analysis determined that Vco-miR166g, Vco-miR166h, and Vco-miR166i precursors share 90%, 90%, and 63% identity with Csi-miR166g, Vvi-MIR166g, and Sly-miR166 b precursors, respectively. We thus concluded that these precursors likely belong to the miR166 family of miRNAs (Table 1). At the same time, these three precursors from highbush blueberry showed conserved features in the 3′ arm and differ by several bases in the 5′ arm with other plant species (Supplementary Figure S2).

From the three pre-miR166s above, we predicted six putative mature miR166 sequences (Table 2), located along the 3′ or 5′ arm of the secondary stem-loop structure of each pre-miR166 sequence by searching against the miRbase database. We designated these candidate miRNAs as Vco-miR166g-3p, Vco-miR166g-5p, Vco-miR166h-3p, Vco-miR166h-5p, Vco-miR166i-3p, and Vco-miR166i-5p. We noticed that the sequence of Vco-miR166i-3p was identical to that of Vco-miR166b-3p, while Vco-miR166i-5p was identical to Vco-miR166d-5p. In addition, BLAST analysis showed that Vco-miR166g-3p, Vco-miR166h-3p, and Vco-miR166i-3p are identical in sequence to known mature miR166s from other species, with the number of mismatches ranging between one (for Vco-miR166g-5p and Vco-miR166h-5p) and two (for Vco-miR166i-5p). These results thus indicated that these new Vco-miR166 candidates do in fact belong to the miR166 family and that the 3′ arm sequences are more conserved than the 5′ arm.

**TABLE 2:** Characteristics of six newly identified mature miR166s in highbush blueberry.

ID	Sequence	Length (nt)	Homologs	Species	NM^a^
Vco-miR166g-3p	UCU​CGG​ACC​AGG​CUU​CAU​UCC	21	Gma-miR166h-3p, Csi-miR166 b,d,g-3p	*Glycine max*, Citrus sinensis	0
Vco-miR166g-5p	GGG​AAU​GCU​GUC​UGG​UUC​GAG	21	Csi-miR166c-5p	Citrus sinensis	1
Vco-miR166h-3p	AUU​UCG​GAC​CAG​GCU​UCA​UUC​C	22	Lja-miR166-3p	Lotus japonicus	0
Vco-miR166h-5p	GGA​AUG​UUG​UCU​GGU​UCG​AGA	21	Csi-miR166c-5p, Zma-miR166g-5p, Bdi-miR166d-5p, Ata-miR166e-5p, Vca-miR166a-5p	Citrus sinensis, *Brachypodium distachyon*, Aegilops tauschii, *Vriesea carinata*	1
Vco-miR166i-3p	UCG​GAC​CAG​GCU​UCA​UUC​CCC	21	Bdi-miR166 b,c,d,i-3p, Gma-miR166c,i-3p, Stu-miR166a,c,d,h-3p, Aly-miR166h-3p, Ata-miR166a,b,d,e-3p, Vca-miR166a,b,c-3p, Eun-miR166-3p, Fve-miR166d-3p, Cas-miR166c,f-3p	*Brachypodium distachyon*, *Glycine max*, *Solanum tuberosum*, *Arabidopsis lyrata*, Aegilops tauschii, *Vriesea carinata*, *Eugenia uniflora*, *Fragaria vesca*, *Camelina sativa*	0
Vco-miR166i-5p	GGG​AUG​UUG​UCU​GGC​UCG​AUG	21	Cly-miR166c-5p, Zma-miR166c-5p, Csi-miR166a,e,f-5p, Aly-miR166a,c,d-5p, Gma-miR166a,c-5p, Osa-miR166d-5p, Mtr-miR166g-5p, Bdi-miR166e-5p, Stu-miR166a-5p, Vca-miR166b-5p, Eun-miR166-5p	Solanum lycopersicum, Zea mays, Citrus sinensis, *Arabidopsis lyrata*, *Glycine max*, Medicago truncatula, *Brachypodium distachyon*, *Solanum tuberosum*, Vriesea carinata, Eugenia uniflora	2

a Number of mismatches between predicted Vco-miR166s, and their homologs.

### Conservation and Diversification of the Vco-miR166 Family

**FIGURE 1:**
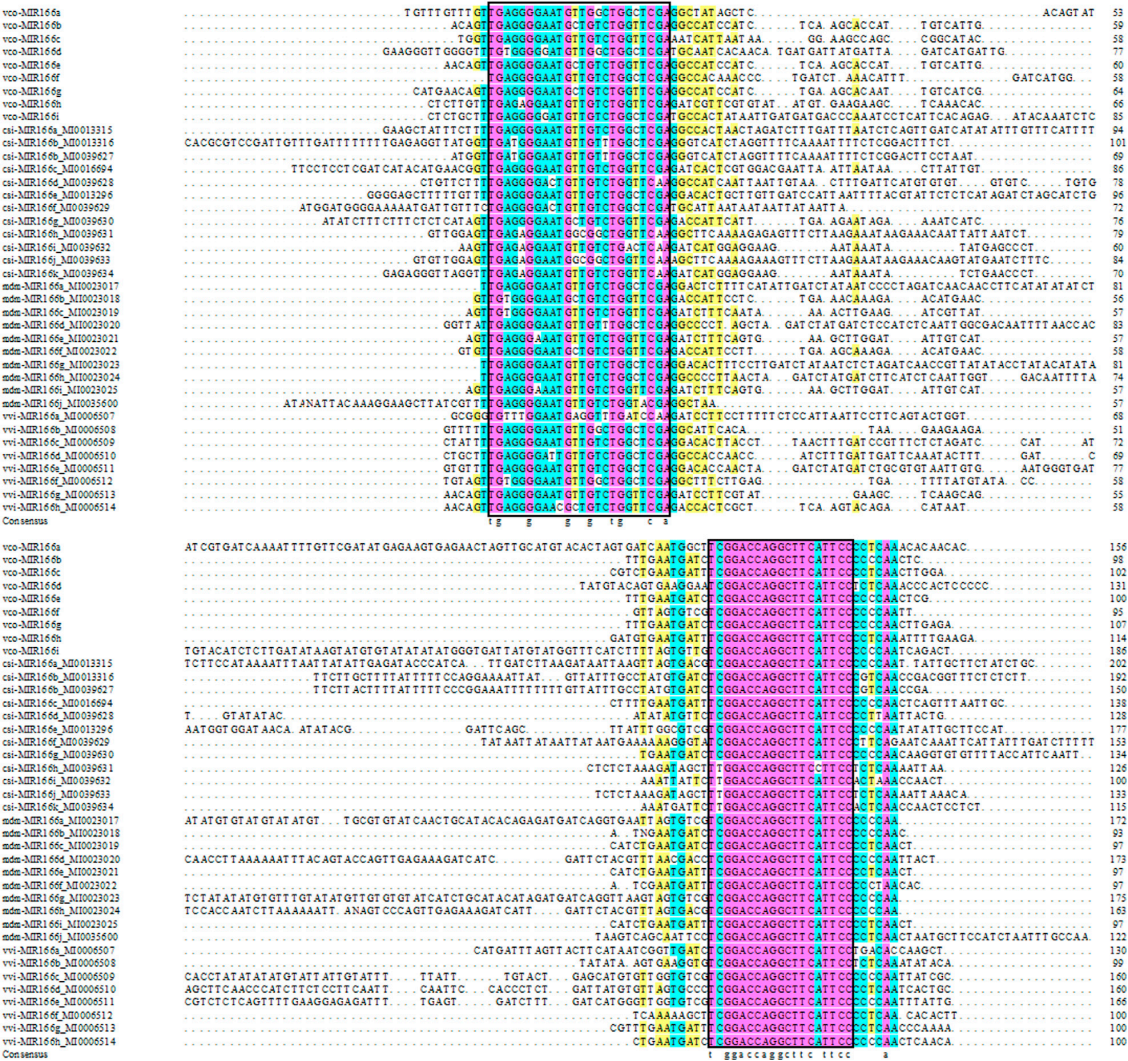
Sequence alignment of miR166 precursors. Vco, *Vaccinium corymbosum*; Csi, *Citrus sinensis*; Vvi, *Vitis vinifera*; Mdm, *Malus domestic*. Pink, 100% conservation; green, ≥75% conservation; yellow, ≥50% conservation. The core region of the mature miRNA is indicated in the black box.

**FIGURE 2:**
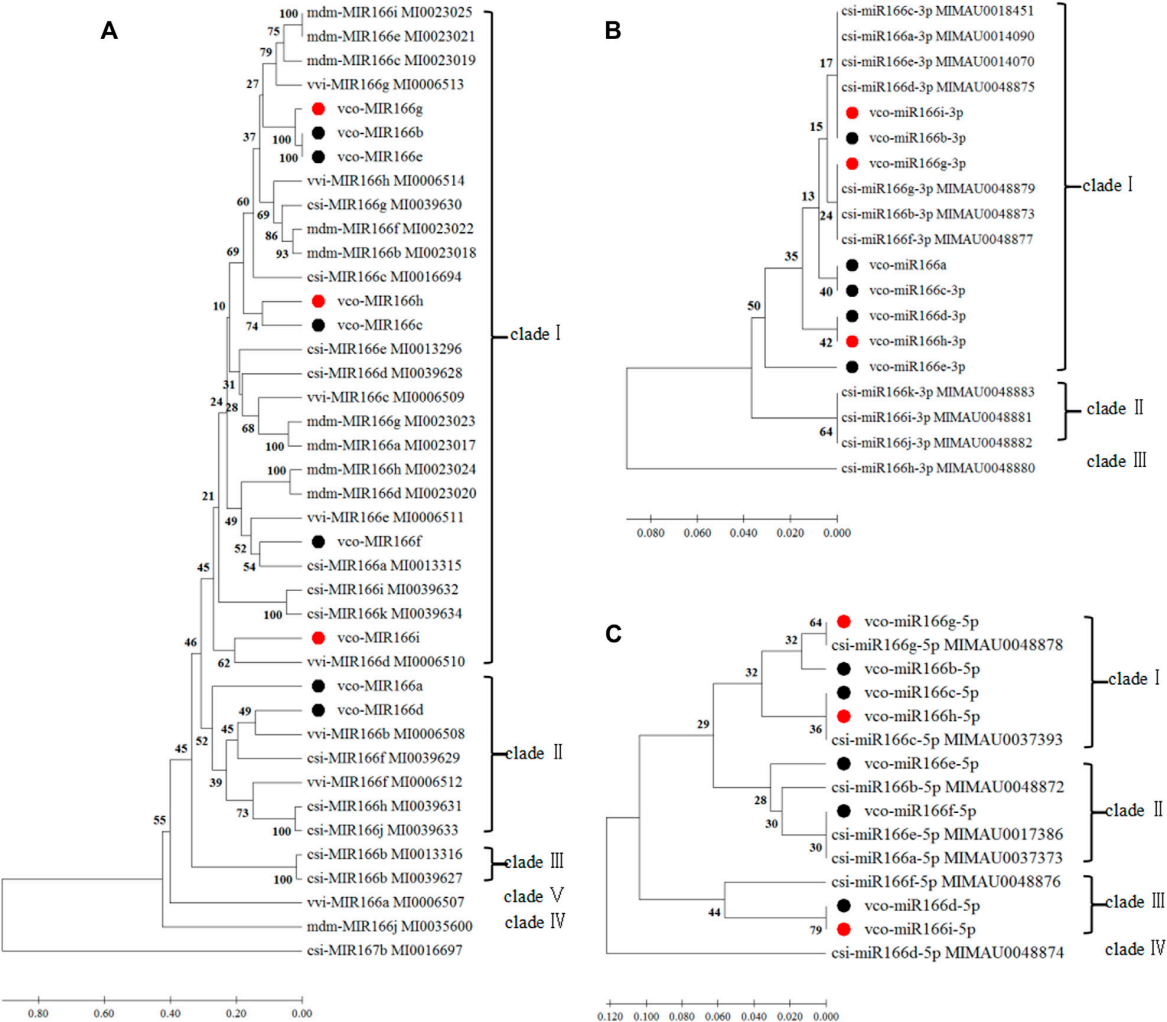
Phylogenetic analyses of *miR166* loci and their predicted 3′ and 5′ arms. Sequences were aligned in MEGA X, and the phylogenetic tree was constructed with the UPGMA method **(A)** Phylogenetic analysis of Vco-miR166 precursors and related sequences from Csi, *Citrus sinensis*; Vvi, *Vitis vinifera* ; and Mdm, *Malus domestica*. Csi-MIR167 b served as the outgroup **(B,C)** Phylogenetic analysis of mature Vco-miR166s and Csi-miR166s for the 3′ arm **(B)** and 5′ arm **(C)**. Black and red dots indicate mature miR166s or their precursors of highbush blueberry from a previous study (Hou et al., 2017) and this study, respectively. The numbers at the nodes indicate the percentage of support from 1,000 bootstrap replicates. The scale indicates the average number of amino acid substitutions per site.

We also aligned the sequences of pre-miR166s from highbush blueberry and other major fruit trees (sweet orange, grape, and apple) and produced the corresponding phylogenetic tree (Figures 1, 2). We observed the highest degree of conservation along the arms of the secondary stem-loop structure, especially on the 3′ arm and in the core region of the mature miRNA (GGA​CCA​GGC​TTC​aTT​CC), with more variation flanking the mature miRNA (Figure 1). The nine Vco-miR166s roughly clustered into clades Ⅰ and Ⅱ but largely did not cluster together within each clade (Figure 2A). These results supported the evolutionary conservation of miR166 family members across various plant species.

We repeated the phylogenetic analysis separately on the 3′ and 5′ arms of nine mature highbush blueberry miR166 sequences along with related sequences from sweet orange (Figures 2B,C). For the analysis of the 3′ arm, Vco-miR166i-3p and Vco-miR166b-3p were identical to Csi-miR166a, c, d, and e-3p, while Vco-miR166g-3p was identical to Csi-miR166b, g, and f-3p in clade I. Eight Vco-miR166s and seven Csi-miR166s clustered into clade I. For the 5′ arm of miR166 sequences, eight Vco-miR166s and eight Csi-miR166s formed four clades, with only clade IV not including a Vco-miR166 member. These results supported the notion that the miR166 family is highly conserved in diverse species. Notably, Vco-miR166e-3p appeared to be distant from other Vco-miR166s based on its position in the phylogenetic tree, indicating the evolutionary diversification of Vco-miR166 members.

### Putative *cis*-Regulatory Elements of *Vco-miR166* Promoters

To explore the potential functions of Vco-miR166s, we analyzed the putative *cis*-regulatory elements contained within 1,600 bp of the *Vco-miR166* promoter sequence upstream of the TSS (+1 bp) (Supplementary Table S2). We identified core promoter elements (TATA-box), common *cis*-acting elements, enhancer regions (CAAT-box), as well as light-, phytohormone-, and defense-related elements in all *Vco-miR166* promoter regions. Several *Vco-miR166* promoters also contained elements related to the cell cycle and meristem expression, seed development, synthesis, and metabolism (Supplementary Table S3). Table 3 summarizes the phytohormone- and defense-related elements in *Vco-miR166* promoters. For plant hormone–related elements, 77.8% (7/9) of *Vco-miR166* promoters harbored gibberellin- and abscisic acid–responsive elements, with 66.7% (6/9) of *Vco-miR166* promoters also presenting elements involved in methyl jasmonate and auxin responsiveness. By contrast, only *Vco-miR166a* and *Vco-miR166h* promoters contained elements associated with salicylic acid responsiveness. For defense-related elements, we detected anaerobic induction elements in all *Vco-miR166* promoter regions, while drought inducibility, low-temperature, and defense- and stress-responsive elements were present in four, three, and two of the nine *Vco-miR166* promoters, respectively. These results indicated that *Vco-miR166* loci may be involved in responses to different phytohormones and defense, but also reflected their potential functional diversification.

**TABLE 3:** Putative *cis*-regulatory elements in upstream sequences of blueberry *Vco-miR166*s.

*Vco-miR166*	Phytohormone-related elements					Defense-related elements			
	Gibberellin responsive	Methyl jasmonate responsiveness	Salicylic acid responsiveness	Auxin responsiveness	Abscisic acid responsiveness	Defense and stress responsiveness	Drought inducibility	Low-temperature responsiveness	Anaerobic induction
*Vco-miR166a*	GARE-motif, P-box	--	TCA-element	--	--	--	MBS	LTR	ARE
*Vco-miR166b*	P-box	TGACG-motif, CGTCA-motif	--	TGA-box, TGA-element	ABRE	--	--	--	ARE
*Vco-miR166c*	--	--	--	AuxRR-core	ABRE	--	--	--	ARE
*Vco-miR166d*	P-box	--	--	TGA-box	ABRE	TC-rich	MBS	--	ARE
*Vco-miR166e*	P-box	TGACG-motif, CGTCA-motif	--	TGA-box, TGA-element	ABRE	--	--	--	ARE
*Vco-miR166f*	GARE-motif	TGACG-motif, CGTCA-motif	--	--	ABRE	--	MBS	--	ARE
*Vco-miR166g*	GARE-motif	TGACG-motif, CGTCA-motif	--	TGA-element, AuxRR-core	ABRE	--	--	LTR	ARE
*Vco-miR166h*	--	TGACG-motif, CGTCA-motif	TCA-element	AuxRR-core	ABRE	--	MBS	LTR	ARE
*Vco-miR166i*	GARE-motif	TGACG-motif, CGTCA-motif	--	--	--	TC-rich	--	--	ARE

TC-rich: cis-acting element involved in defense and stress responsiveness; MBS: MYB, binding site involved in drought-inducibility; LTR: cis-acting element involved in low-temperature responsiveness; ARE: cis-acting regulatory element essential for the anaerobic induction.

### Prediction of Vco-miR166 Target Genes

To obtain a better understanding of Vco-miR166 functions, we predicted their targets with the psRNATarget program against the transcript database for American cranberry (*V. macrocarpon*) transcripts, another *Vaccinium* species, because there is no highbush blueberry database in the psRNATarget program (Supplementary Table S4 **)**. We then used the American cranberry targets to search for related sequences in the highbush blueberry (*V. corymbosum* cv. Draper v1.0) transcripts (Supplementary Table S5). All Vco-miR166s were predicted to target the transcripts of a gene encoding a pentatricopeptide repeat–containing protein (Supplementary Table S6). In addition, all Vco-miR166s except Vco-miR166h-3p were predicted to target the transcripts encoding a leucine-rich repeat (LRR) receptor-like serine/threonine-protein kinase. Many Vco-miR166s-3p were predicted to regulate genes encoding homeobox-leucine zipper proteins (HD-ZIP III), while many Vco-miR166s-5p were predicted to target the transcripts for genes encoding phosphatidylinositol/phosphatidylcholine transfer proteins and patellin. These results supported the functional conservation of Vco-miR166 family members. Several Vco-miR166s involved in abiotic stresses, including heat, stress response, and cold, were predicted to target transcripts for cold-regulated proteins, while other Vco-miR166s appeared to target the transcripts encoding many protein families. For example, Vco-miR166a,b,i-3p targeted ethylene-responsive and basic helix-loop-helix (bHLH) transcription factors, Vco-miR166c-3p targeted WD repeat–containing protein, Vco-miR166d-3p targeted bHLH transcription factors, and Vco-miR166e,g-3p targeted WRKY transcription factors. These results illustrated the functional diversification of Vco-miR166 family members in highbush blueberry.

To explore the diversity of Vco-miR166 functions, we calculated the genetic similarity and genetic distance according to the presence or absence of functions of their target genes in Supplementary Table S6. The genetic similarity was the highest (0.88) and the genetic distance was the closest (0.12) between Vco-miR166a-3p and Vco-miR166b/i-3p, whereas Vco-miR166b/i-3p and Vco-miR166b-5p exhibited the lowest genetic similarity (0.05) and the farthest genetic distance (3.03) (Table 4). The genetic similarity between Vco-miR166h-3p and Vco-miR166i-3p was also low at only 0.12. In fact, a phylogenetic tree according to the genetic similarity between miR166 targets showed that Vco-miR166s are divided into two clades, with Vco-miR166s derived from the same arm clustering together (Supplementary Figure S3). These results indicated that the functions of Vco-miR166s are diverse and that Vco-miR166s from the same arm exert similar regulatory functions.

**TABLE 4:** Genetic distance and genetic similarity between Vco-miR166s from highbush blueberry according to the functions of target genes.

	Vco-miR166a	Vco-miR166b/i-3p	Vco-miR166c-3p	Vco-miR166d-3p	Vco-miR166e-3p	Vco-miR166g-3p	Vco-miR166h-3p	Vco-miR166b-5p	Vco-miR166c-5p	Vco-miR166d/i-5	Vco-miR166e-5p	Vco-miR166f-5p	Vco-miR166g-5p	Vco-miR166h-5p
Vco-miR166a		0.12	1.27	0.31	1.23	1.66	1.28	2.91	2.28	2.56	2.85	2.63	2.83	2.57
Vco-miR166b/i-3	0.88		1.28	0.24	1.15	1.67	1.30	3.03	2.40	2.68	2.97	2.76	2.95	2.70
Vco-miR166c-3p	0.28	0.27		1.02	1.61	1.92	1.66	2.48	2.25	2.94	2.41	2.60	2.40	2.03
Vco-miR166d-3p	0.73	0.78	0.36		1.19	1.62	1.04	2.86	2.01	2.52	2.80	2.59	2.79	2.24
Vco-miR166e-3p	0.29	0.31	0.20	0.31		0.59	1.81	2.15	1.65	1.99	2.09	1.72	2.36	2.22
Vco-miR166g-3p	0.18	0.19	0.14	0.19	0.53		2.12	1.37	1.61	1.96	1.71	1.49	2.16	1.62
Vco-miR166h-3p	0.28	0.27	0.19	0.35	0.16	0.12		2.90	2.26	2.55	2.83	2.33	2.82	2.56
Vco-miR166b-5p	0.05	0.05	0.08	0.06	0.11	0.25	0.05		1.57	1.57	0.82	0.80	0.74	1.01
Vco-miR166c-5p	0.10	0.09	0.10	0.13	0.19	0.20	0.10	0.21		1.53	1.91	1.33	1.90	1.13
Vco-miR166d/i-5	0.08	0.07	0.05	0.08	0.13	0.14	0.08	0.21	0.22		1.51	0.96	2.08	1.44
Vco-miR166e-5p	0.06	0.05	0.09	0.06	0.12	0.18	0.06	0.44	0.15	0.22		0.89	1.49	1.52
Vco-miR166f-5p	0.07	0.06	0.07	0.07	0.17	0.22	0.09	0.45	0.26	0.38	0.40		1.28	0.97
Vco-miR166g-5p	0.06	0.05	0.09	0.06	0.09	0.11	0.06	0.47	0.15	0.12	0.23	0.27		1.22
Vco-miR166h-5p	0.08	0.07	0.13	0.10	0.11	0.20	0.08	0.36	0.32	0.24	0.22	0.38	0.29	

Genetic distances are indicated in the upper right triangle; genetic similarity is shown in the lower left triangle.

### Accumulation of Vco-miR166s Under Abiotic Stress

**FIGURE 3:**
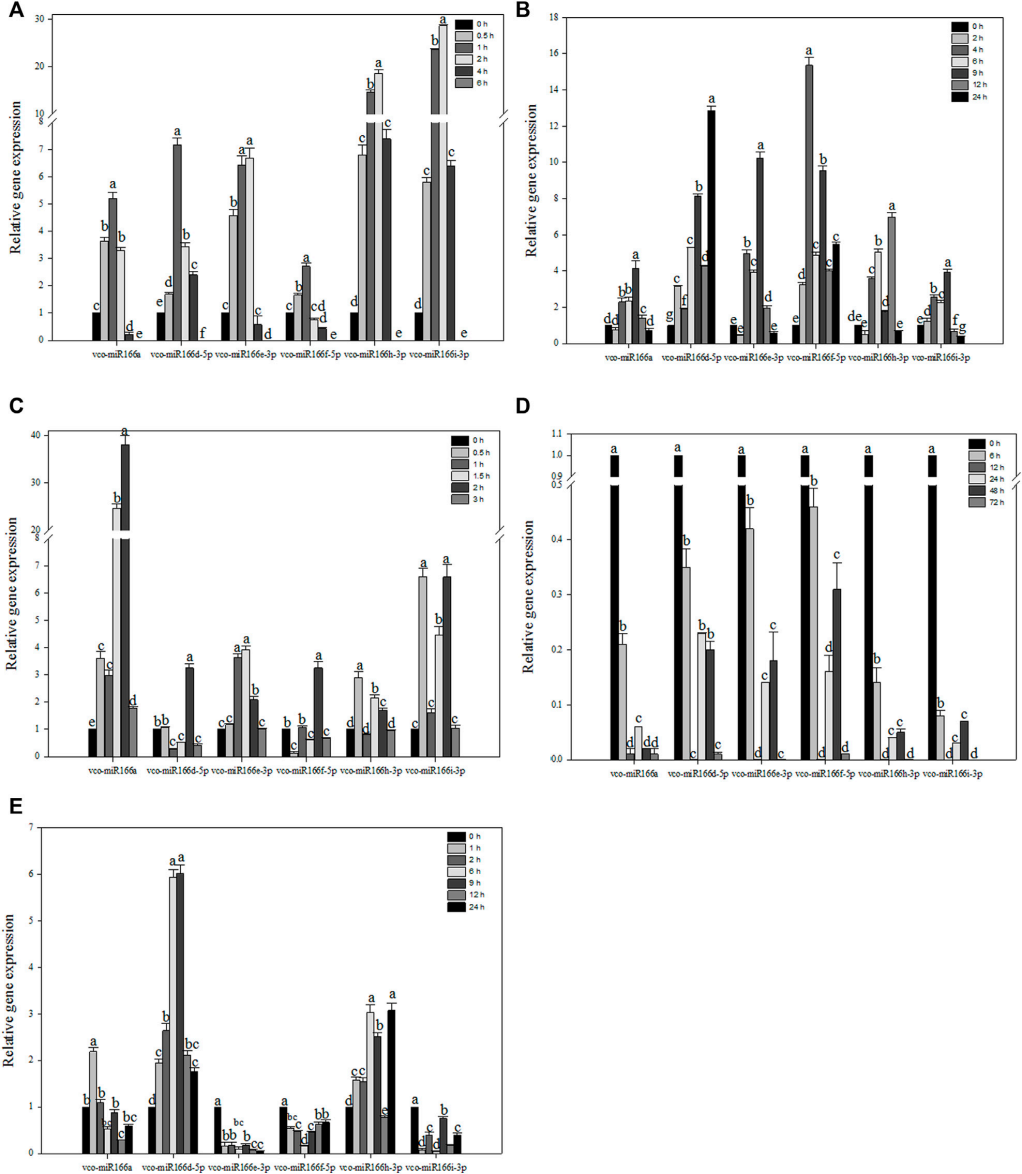
RT-qPCR analysis of Vco-miR166 abundance under various stress conditions. Highbush blueberry plants were exposed to freezing **(A)**, cold **(B)**, heat **(C)**, drought **(D)**, and salt **(E)** stress. Data represent the means ± standard deviation (SD) of three independent biological replicates, each with three technical replicates. Different letters indicate significant differences (*p* < 0.05) between samples, as determined by Tukey’s test.

To explore how Vco-miR166s are related to abiotic stress, we selected six Vco-miR166s to examine their abundance in response to abiotic stresses (Figure 3). The six Vco-miR166s had similar accumulation patterns under freezing stress, as they all rapidly increased in abundance, with Vco-miR166h-3p and Vco-miR166i-3p increasing in abundance by 18.6- and 28.6-fold upon 2 h of freezing stress compared to the control at 0 h. The abundance of the six Vco-miR166 transcripts sharply declined and showed no accumulation after 6 h of cold exposure in 1-year-old blueberry plants, which died after 6 h of –5°C treatment (Figure 3A). These results suggested that these six Vco-miR166s, especially Vco-miR166h-3p and Vco-miR166i-3p, may be involved in freezing response and as negative regulators.

In blueberry plants exposed to cold stress, the abundance of Vco-miR166a, Vco-miR166e-3p, and Vco-miR166i-3p reached their maximum levels after 9 h of freezing stress, corresponding to an increase of 4.1-, 10.2-, and 3.9-fold compared to the control at 0 h, respectively, followed by a sharp decrease to control levels for Vco-miR166a and Vco-miR166e-3p or to lower levels than control for Vcc-miR166i-3p. The abundance of Vco-miR166d-5p, Vco-miR166f-5p, and Vco-miR166h-3p was upregulated and reached its highest levels at 24, 4, and 12 h during freezing stress, increasing 12.8-, 15.4-, and 8.5-fold compared to the control samples, respectively (Figure 3B). Thus, the Vco-miR166 family appeared to be involved in cold stress responses.

When blueberry plants were exposed to heat stress, Vco-miR166a precursor abundance was upregulated and increased by 38.1-fold at 2 h compared to the control samples at 0 h, followed by a sharp decrease at 3 h. Vco-miR166d-5p and Vco-miR166f-5p showed similar accumulation patterns, with their levels reaching their peak at 2 h and their lowest levels at 1.5 and 3 h. The accumulation of Vco-miR166e-3p, Vco-miR166h-3p, and Vco-miR166i-3p was also upregulated and reached its highest levels at 0.5 h for Vco-miR166h-3p, at 1 and 1.5 h for Vco-miR166e-3p, and at 0.5 and 2 h for Vco-miR166i-3p. All six miRNAs showed a rapid decrease in abundance at 3 h (Figure 3C). Therefore, Vco-miR166 family members might respond during early stages of heat stress.

Under drought stress, six Vco-miR166 precursors showed similar trends in their abundance, with a sharp decrease and reaching their lowest levels at 12 and 72 h and a modest increase in their levels at 24 and 48 h (Figure 3D). Vco-miR166 members might thus promote the accumulation of their target transcripts and play a positive role in stress adaptation.

In the context of salt stress, Vco-miR166a transcript levels were upregulated after 1 h and then rapidly decreased, with the lowest abundance after 12 h. We observed similar patterns for Vco-miR166d-5p, whose transcript levels gradually increased by 6.9- and 6.0-fold at 6 and 9 h compared to the 0 h control and then rapidly increased until 24 h. Vco-miR166h-3p also increased in abundance before decreasing, but increased rapidly at 24 h. On the contrary, the transcript levels of Vco-miR166e-3p, Vco-miR166f-5p, and Vco-miR166i-3p were downregulated compared to the control under salt stress, especially Vco-miR166e-3p, whose transcript levels rapidly decreased at 1 h and then remained low after salt stress (Figure 3E). These results suggested that these Vco-miR166 members might respond to salt stress at an early stage.

## Discussion

miRNAs play various roles in regulating almost all processes of plant growth and in metabolism. Several conserved miRNAs were reported in highbush blueberry based on an analysis of expressed sequence tags (ESTs) (Li et al., 2014), leading to the identification of 10 miR166 family members using high-throughput sequencing of small RNAs and reference sequences from the American cranberry genome, and ESTs and transcriptome data from blueberries during fruit maturation (Hou et al., 2017). The completion of the highbush blueberry genome (Gupta et al., 2015) allowed us to identify three new *Vco-pre-miR166* members and determine their mature miR166s based on the *V. corymbosum* cv. Draper v1.0 genome. We determined here that the newly identified pre-miR166s exhibit low MFE and high MFEI values and form stable secondary hairpin structures (Table 1 and Supplementary Figure S1). In addition, we showed that the mature Vco-miR166 sequences are highly identical to other miR166 family members from other species (Table 2). We thus concluded that the three newly identified *Vco-miR166* loci belong to the miR166 family.

The conserved nature of *miR166* family members has been demonstrated in many plant species (Li et al., 2017; Barik et al., 2014). In this study, we showed that the newly identified mature Vco-miR166s derived from the 3′ and 5′ arms have an identical sequence and differ by one or two bases with mature miR166s from other plant species (Table 2). Phylogenetic analysis indicated that some Vco-miR166s are identical to mature Csi-miR166s, while the precursor sequences of miR166s from blueberry, sweet orange, grape, and apple also showed a high degree of conservation along the 3′ arm (Figure 1). Thus, miR166 family members are highly conserved across diverse plant species, especially along the 3′ arm (Barik et al., 2014). Mature miR166 members are known to regulate the abundance of transcripts encoding HD-ZIP III transcription factors in many species (Boualem et al., 2008; Li R. et al., 2018; Yadav et al., 2021). In this study, we predicted that all Vco-miR166s derived from the 3′ arm target HD-ZIP III transcripts, supporting the functional conservation of miR166 family members in various species (Supplementary Table S6).

The multiple *miR166* family members likely diverged from a common ancestor, as reflected by the evolutionary diversification of the miR166 precursor sequences (Barik et al., 2014; Li et al., 2017). In addition, an analysis of the *Vco-miR166* promoters and the putative Vco-miR166 target genes indicated that Vco-miR166 exert diverse functions (Li et al., 2017). Indeed, we detected different defense-related elements and anaerobic induction elements in the promoters of *Vco-miR166* loci (Table 3). The prediction of Vco-miR166 target genes showed that most Vco-miR166s target different genes (Table 4). These results suggest the functional diversification of Vco-miR166s, in agreement with similar results obtained in other plant species (Barik et al., 2014; Li et al., 2017).

Temperature is an important environmental factor that affects plant growth, yield, and geographical distribution. Plants are constantly subjected to freezing, cold, and heat stress from their surrounding natural environment, with miRNAs playing an active role in temperature stress responses (Sun et al., 2019; Fang and Wang, 2021). In rice (*Oryza sativa*), Osa-miR166 m and Osa-miR166 k are involved in cold stress (Devi et al., 2013). Similarly, Ath-miR166 abundance is upregulated in Arabidopsis seedlings exposed to cold stress (Zhou et al., 2008), while Stu-miR166 (from tomato) and miR166a-e [from almond (*Prunus dulcis*)] abundance decreased under cold stress (Ou et al., 2015; Karimi et al., 2016). In tomato, upregulation of Sly-miR166 and the concomitant downregulation of its target gene encoding an HD-ZIP III showed that Sly-miR166 plays a critical role in regulating cold stress responses in this species (Valiollahi et al., 2014). Under chilling conditions, miR166e-3p abundance in field mustard (*Brassica rapa*) was upregulated, while miR166a levels were downregulated; in almond, the abundance of miR166a-e decreased upon exposure to cold (Zeng et al., 2018; Karimi et al., 2016). In the context of heat exposure, miR166 abundance decreased in radish (*Raphanus sativus*) but increased in rice (Wang et al., 2015; Li et al., 2014a). However, miR166a/b/c-3p abundance increased while that of miR166c/f-5p decreased in *Betula luminifera* under heat stress (Pan et al., 2017). The abundance of almost all Vco-miR166s characterized here transiently increased before dropping under temperature stress. In particular, the levels of Vco-miR166s rapidly increased within 1 or 2 h under freezing stress (Figure 3A). At the same time, the promoters of several *Vco-miR166*s (*Vco-miR166a*, *Vco-miR166g*, and *Vco-miR166h*) harbored a low-temperature response element; notably, Vco-miR166s also targeted transcripts encoding HD-Zip III transcription factors, which are involved in temperature stress (Valiollahi et al., 2014). The functions of predicted target genes showed that Vco-miR166f-5p and other Vco-miR166s may target heat and cold shock proteins and directly regulate temperature stress responses (Supplementary Table S6). Our results indicated that Vco-miR166s may be early positive regulators of the response to temperature stress.

Drought is another major abiotic stress that limits plant growth and development. Many miRNAs regulate drought responses in plants (Li et al., 2013; Budak et al., 2015; Pagliarani et al., 2017). Indeed, miR166 expression was shown to be downregulated in barley and in emmer wheat, while being upregulated in rubber tree (*Hevea brasiliensis*) in response to drought (Kantar et al., 2010; Kuruvilla et al., 2019; Kantar et al., 2011). In rice, manipulating the levels of miR166 demonstrated its contribution to drought resistance (Zhang et al., 2018). In white mulberry (*Morus alba multicaulis*), miR166f might function as a positive regulator of drought stress (Li R. et al., 2018). In this study, we showed that the abundance of all Vco-miR166s decreases under drought stress. Thus, the Vco-miR166 family might act as a negative regulator of drought stress.

Salt stress is a major abiotic stress that affects plant growth and development. Many miRNAs, including miR166 family members, play important roles in response to salt stress in plants (Si et al., 2014; Sun et al., 2019; Fang and Wang, 2021). The abundance of miR166a-5p and miR166j-3p was upregulated in soybean roots, while that of miR166a-5p was downregulated in barley roots (Wang et al., 2020; Kuang et al., 2021). However, miR166 family members were upregulated in roots and downregulated in leaves of sweet potato (*Ipomoea batatas*) (Yang et al., 2020), while Pgu-miR166t was also downregulated in the leaves of common guava (*Psidium guajava*) (Sharma et al., 2020). In our study, the levels of Vco-miR166e-3p, Vco-miR166f-5p, and Vco-miR166i-3p were downregulated in highbush blueberry leaves, while those of Vco-miR166d-5p and Vco-miR166h-3p were upregulated upon exposure to salt stress relative to controls. However, Vco-miR166a levels rose at 1 h and dropped at 12 h following exposure to salt stress (Figure 3E). These results indicated that different members of the Vco-miR166 family exhibit distinct expression patterns and form a complex regulatory network under salt stress.

Here, we identified three new *miR166* loci in the highbush blueberry genome, which increased the size of the highbush blueberry miR166 family. The resulting 16 *Vco-miR166* members of highbush blueberry exhibited evolutionary conservation and functional diversification based on sequence analysis, promoter dissection, and target gene predictions. The analysis of their upstream sequences and stem-loop RT-qPCR showed that Vco-miR166 family members are involved in various abiotic stresses, possibly by positively and/or negatively regulating target genes.

## Data Availability

The original contributions presented in the study are included in the article/Supplementary Material, further inquiries can be directed to the corresponding author.
