# Supplementary material for: Conservation and Diversity of miR166 Family Members From Highbush Blueberry (Vaccinium corymbosum) and Their Potential Functions in Abiotic Stress
Source: Front Genet. 2022 May 16;13:919856. doi: 10.3389/fgene.2022.919856 (PMC9149266; doi:10.3389/fgene.2022.919856)
Supplement: Supplementary file 2 [file Image3.pdf]

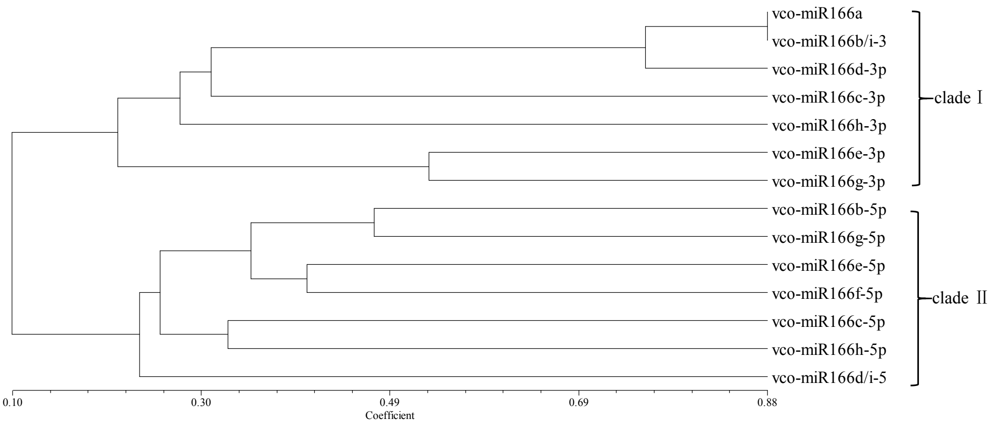

Supplementary Figure 3 | Phylogenetic relationship of Vco-miR166s according to the functions of their predicted target genes. The phylogenetic tree was constructed with the UPGMA method based on DICE similarity coefficient. The DICE similarity coefficients were calculated according to the binary matrix, where 0 and 1 coded for absence and presence of functions of their target genes, respectively.
