# Supplementary material for: Conservation and Diversity of miR166 Family Members From Highbush Blueberry (Vaccinium corymbosum) and Their Potential Functions in Abiotic Stress
Source: Front Genet. 2022 May 16;13:919856. doi: 10.3389/fgene.2022.919856 (PMC9149266; doi:10.3389/fgene.2022.919856)
Supplement: Supplementary file 3 [file Image1.pdf]

Pre-Vco-miR166g

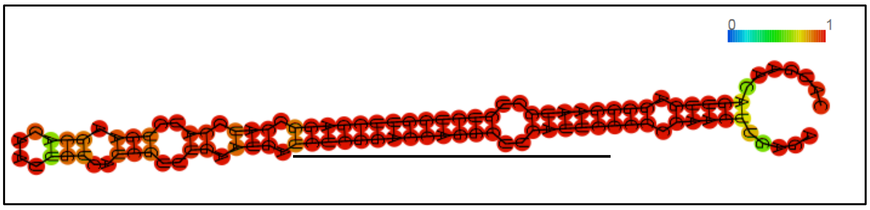

Pre-Vco-miR166h

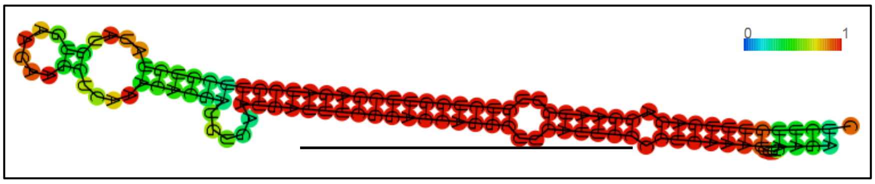

Pre-Vco-miR166i

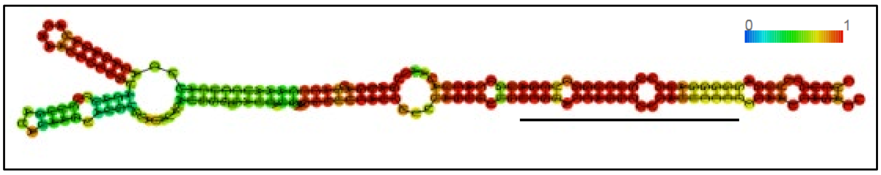

Supplementary Figure 1 | Stem-loop structures of three newly identified pre-miR166s from highbush blueberry. The mature miRNA portion is indicated by the black horizontal line. The gradation from blue to red represents the base-pair probabilities from 0 to 1.
