# Supplementary material for: Conservation and Diversity of miR166 Family Members From Highbush Blueberry (Vaccinium corymbosum) and Their Potential Functions in Abiotic Stress
Source: Front Genet. 2022 May 16;13:919856. doi: 10.3389/fgene.2022.919856 (PMC9149266; doi:10.3389/fgene.2022.919856)
Supplement: Supplementary file 1 [file Image2.pdf]

```
                                   5' arm
Vco-miR166g   1    ACAGUUGAGGGGAAUGCUGUCUGGUUCGAGGCCAUCCAUCUGAAGCACAAUUGUCAUCGU
                   ||||||||||||| |||||||||||||||| | |||||| |||||  |||    ||||||
Csn-miR166g   1    ACAGUUGAGGGGAGUGCUGUCUGGUUCGAGACUAUCCAUUUGAAGUGCAAAC..CAUCGU

Vco-miR166g   61   UUGAAUGAUCUCGGACCAGGCUUCAUUCCCCCAACUUGA
                    ||||||||||||||||||||||||||||||||||||||
Csn-miR166g   59   CUGAAUGAUCUCGGACCAGGCUUCAUUCCCCCAACUUGA
                                  3' arm

                               5' arm
Vco-miR166h   1    UUGAGAGGAAUGUUGUCUGGUUCGAGAUCGUUCGUGUAUAUGUGAAGAAGCUCAAACACG
                   ||||| ||||||||||||||||||||||| |||||         | ||||||||| ||
Vvi-miR166g   1    UUGAGGGGAAUGUUGUCUGGUUCGAGAUCCUUCGU.........AUGAAGCUCAAGCAGC

Vco-miR166h   61   AUGUGAAUGAUUUCGGACCAGGCUUCAUUCCCCUCAA
                    | ||||||||||||||||||||||||||||||||||
Vvi-miR166g   52   GUUUGAAUGAUUUCGGACCAGGCUUCAUUCCCCUCAA
                               3' arm

                                       5' arm
Vco-miR166i   1    CUCUGCUUUGAGGGGGAUGUUGUCUGGCUCGAUGCCACUAUAAUUGAUGAUGACCCAAAU
                   ||||   |||||||| |||||||||||||||| | ||| |         || | ||  ||
Sly-miR166b   1    CUCUCAGUUGAGGGGAAUGUUGUCUGGCUCGAGGUCACCAAUUAGAUCCAUAAUCCCUAU

Vco-miR166i   61   CCUCAUUCACAGAGAUACAAAUCUCUGUACAUCUCUUGAUAUAAGUAUGUGUAUAUAUAU
                                              || | |      |  ||  |   |  |    ||
Sly-miR166b   61   A..........................UAUACCAUGCUUUUCAAAAAAAAGAUUUAUAAU

Vco-miR166i   121  GGGUGAUUAUGUAUGGUUUCAUCUUUUAGUGUUGUCGGACCAGGCUUCAUUCCCCCAAU
                     |  |    ||   |  |||| | ||||||||||||||||||||||||||||||||||
Sly-miR166b   95   AUGAAAAGGGGUUAUGAAUCAUGUGUUAGUGUUGUCGGACCAGGCUUCAUUCCCCCAAU
                                                            3' arm
```

Supplementary Figure 2 | The sequence alignments of miR166 precursors between highbush blueberry and other species
